# Prognostic scoring systems in chronic myeloid leukaemia

**DOI:** 10.1038/s41375-025-02606-6

**Published:** 2025-04-15

**Authors:** Michael Lauseker, Verena S. Hoffmann, Markus Pfirrmann

**Affiliations:** Institute for Medical Information Processing, Biometry, and Epidemiology, Faculty of Medicine, Ludwig-Maximilians-Universität München, Munich, Germany

**Keywords:** Risk factors, Chronic myeloid leukaemia

## Abstract

Prognostic scores are an important tool in medical statistics. In chronic myeloid leukaemia (CML), prognostic models have existed for many years, enabling the classification of patients into groups that can be clearly differentiated in terms of their prognosis. However, over time, the focus of these models has shifted from solely survival outcomes to a broader range of diverse endpoints. This review explores the development and applications of these scores, offering recommendations for their use, and looks ahead to potential future advancements in the field. As the landscape of CML treatment evolves with newer therapeutic options, it is crucial to adapt prognostic models to reflect not only survival rates but also other important clinical milestones such as molecular remission, progression-free survival, and CML-related survival. The continued refinement of these tools, alongside international validation efforts, will be essential in providing clinicians with more accurate and individualized patient prognostication, ultimately improving therapeutic decision-making and patient outcomes.

## Introduction

Prognostic research is a crucial field in medical statistics, as it helps improve the prediction of future disease progressions and treatment outcomes. By analysing patient data, genetic markers, and clinical factors, statistical models can be developed to predict the risk of specific disease trajectories or responses to therapies. These insights enable a more individualized and precise approach to medicine, allowing physicians to make informed decisions and select the best possible treatment for each patient.

Patient characteristics that exhibit a statistically significant and clinically relevant association with a clearly defined future disease outcome are referred to as prognostic factors. When multiple factors are considered together, they can be combined into a prognostic score. This score is derived from a mathematical formula, where each factor is assigned a specific weight and, if necessary, transformed—often using a regression model or a similar method. The prognostic score is then used to classify patients into two or more prognostic groups based on predefined rules. To ensure these groups are meaningful, they must demonstrate clinically relevant differences in their predicted outcomes. The combination of the prognostic score and the classification rules is generally referred to as a “prognostic model.” However, in the context of chronic myeloid leukaemia (CML), the term “score” has become more common [1], and this terminology will also be used here.

Prognostic scores provide an estimate of the course of a disease but do not allow for the assessment of the effect of a specific therapy. This distinguishes prognostic scores from predictive scores, which are used to estimate the effect of a treatment [2]. Unlike other diseases, risk scores in CML that focus on the risk factors for its occurrence, to our knowledge, do not exist. This work is therefore largely limited to classical prognostic scores. However, the term “risk scores” has become widely established, so in the following, the term “risk scores” is used, even when prognostic scores are actually meant.

Prognostic scores have numerous applications. One important example is their use for the individualized prediction of outcomes. This enables the treating physician to determine appropriate intervals for follow-up examinations. Additionally, knowing the predicted outcome helps weigh the effectiveness of a treatment against its potential side effects when making therapeutic decisions. Beyond the individual patient, prognostic scores are also crucial for characterizing patient populations. This is particularly important when comparing study results, as adjustments for differing risk profiles are necessary, or when using these scores as stratification factors in randomized clinical trials.

## Development of life expectancy in CML

Compared to the current situation, the prognosis for CML was historically very poor. As recently as the 1980s and 1990s, median survival in clinical studies was approximately five years [3]. Treatment options at the time were limited. Nevertheless, even in that era, some patients achieved survival times of ten or even twenty years [3]. This significant heterogeneity in outcomes highlighted the need to identify these long-term survivors, leading to the development of the first prognostic systems during that period. The advent of targeted therapies, specifically tyrosine kinase inhibitors (TKIs), has brought remarkable improvements over the past two decades. Early studies on imatinib quickly demonstrated that it represented a major medical breakthrough. Ten-year survival rates for these patients exceeded 80% [4, 5]. Over time, further therapeutic refinements and the introduction of additional TKIs with the option to save patients that failed under imatinib led to modest but meaningful improvements in survival. Recently, a 95% overall survival after 8 years was published for the German TIGER study [6]. In clinical trial settings, CML patients now generally have a life expectancy close to that of the general population [7]. Outside of clinical trials, particularly in developed countries, life expectancy for CML patients has also approached that of the general population [8–10]. Today, most CML patients are expected to die from causes unrelated to their leukaemia. However, in less developed regions, limited access to these therapies remains a significant issue, resulting in considerably lower life expectancy for patients in these areas [11].

## Prognostic scores

Numerous scoring systems exist for CML. When applying these systems, three key factors must be considered: for which endpoint they were developed, for which patient cohort, and at what time point they are applied.

Early prognostic research in CML naturally focused on overall survival as the primary endpoint. This endpoint is of undeniable relevance to patients and was thus a logical choice. However, since the advent of TKIs, progression to advanced phase and in consequence deaths from CML, have become rare. Consequently, developing a score now requires either a very large sample size or the use of a more frequently occurring endpoint. Alternative endpoints in prognostic research include composite surrogate parameters such as progression-free survival (PFS) or failure-free survival (FFS). For progression-free survival, it is important to account for the competing definitions of CML phases that have emerged over time [12, 13]. In the case of failure-free survival, the definition is even more heterogeneous, and it combines endpoints that vary significantly in their importance to patients and in how frequently they are recorded [14]. However, the advantage of composite endpoints lies in the increased number of events available for analysis.

Given that more CML patients are dying from causes unrelated to CML, recent studies have taken the opposite approach by focusing on CML-related survival. This requires cohort studies with relatively large sample sizes (and an adequate data quality) but ensures that only deaths attributable to CML are considered, e.g. when the patient progressed to blast phase before death.

Other potential endpoints include the achievement of remission, either over time or at a specified milestone. In recent years, deep molecular remissions have become a focal point, especially given the goal of treatment-free remission (TFR). In earlier years, however, hematologic or cytogenetic remissions were more prominent. For predicting the stability of TFR, the loss of remission serves as a natural endpoint.

After selecting the endpoint, it is essential to consider the underlying patient cohort. While many prognostic models can be applied to similar scenarios, their performance may vary: A model developed e.g., for adult patients might or might not perform well in children. In general, it can still be assumed that prognostic models will yield similar results under closely related therapies. However, a model developed for patients treated with interferon will no longer produce accurate predictions when applied to modern therapy options with third-generation TKIs. The cohort’s therapeutic context also matters. Although prognostic models are not designed to guide treatment decisions, the predictive value of the included factors may vary across different therapies.

Finally, the timing of the model’s application is crucial. Prognostic models are typically designed based on a defined milestone, such as the diagnosis of CML. To ensure validity, the same variables must be used at the corresponding time point, particularly in the absence of prior therapy. While dynamic prognostic models that continuously adjust throughout the disease course are statistically feasible, they are not yet widely implemented. This may be partly due to the success of current therapies, which often do not necessitate urgent adjustments. Consequently – if not aiming for treatment-free remission - monitoring for patients with favourable responses is often done with reduced frequency [15].

## Scores in CML

The development of prognostic scoring systems in chronic myeloid leukaemia reflects advancements in therapy and shifting treatment goals over the decades. The Sokal score [16], developed in the early 1980s, was the first widely adopted scoring system. It was created using data from 678 patients in the chronic phase at diagnosis who were treated with chemotherapy. The score categorized patients into three roughly equal risk groups but struggled to adequately distinguish [17] between intermediate- and high-risk groups clinically. It uses the following parameters: age, spleen size, platelet count, and percentage of blasts in peripheral blood. A summary on all the scores discussed in this section can be found in Table 1.

**Table 1:** Scores discussed in this review.

Score	Formula	Risk definition	Distribution^a^	Reference
Sokal [16]	exp(0.0116 x (age –43.4) + 0.0345 x (spleen – 7.51) + 0.1880 x [(platelet count/700)²−0.563] + 0.0887 x (blasts – 2.10))	Low risk <0.8 Intermediate risk: 0.8−1.2 High risk: >1.2	Low risk: *n *= 114, 32% Intermediate risk: *n *= 145, 40% High risk: *n *= 102, 28%	PMID: 6584184
Euro [17]	(0.6666 [if age ≥50] + 0.0420 x spleen + 0.0584 x blasts + 0.0413 x eosinophils + 0.2039 [if basophils ≥ 3] + 1.0956 [if platelet count ≥1500 ×10^9^/L]) x 1000	Low risk ≤780 Intermediate risk: >780–1480 High risk: >1480	Low risk: *n *= 369, 41% Intermediate risk: *n *= 406, 45% High risk: *n *= 133, 15%	PMID: 9625174
EUTOS [19]	basophils x 7 + spleen x 4	Low risk: ≤87 High risk: >87	Low risk: *n *= 836, 90% High risk: *n *= 90, 10%	PMID: 21536864
ELTS [24]	0.0025 x (age/10)³ + 0.0615 x spleen + 0.1052 x blasts + 0.4104 x (platelet count/1000)^−0.5^	Low risk ≤1.5680 Intermediate risk: >1.5680–2.2185 High risk: >2.2185	Low risk: *n *= 1349, 61% Intermediate risk: *n *= 596, 27% High risk: *n *= 260, 12%	PMID: 26416462
IMTF [33]	1 [if ELTS = intermediate risk] + 2 [if ELTS = high risk] + 1 [if WBC ≥ 120 ×10^9^/L] + 1 [if Haemoglobin < 115 g/L] + 1 [if basophils ≥ 12%]	Very low risk: 0 Low risk: 1 Intermediate risk: 2 High risk: 3 Very high risk: ≥4	Very low risk *n *= 315, 35% Low risk: *n *= 180, 20% Intermediate risk: *n *= 192, 21% High risk: *n *= 147, 16% Very high risk: *n *= 74, 8%	PMID: 35194158
MMRscore [36]	−0.3814 [if sex = female] – 0.1683 x (WBC/100) + 0.7201 x (haemoglobin/100) – 0.0861 x (blasts + 1) – 0.3775 x [(spleen + 0.1) /10]	Low risk ≥0.3007 Intermediate risk: ≥ −0.8505–<0.3007 High risk: ≤ −0.8505	Low risk: *n *= 344, 40% Intermediate risk: *n *= 390, 46% High risk: *n *= 119, 14%	PMID: 35650426
MR^4^score [36]	−0.2834 [if sex = female] – 0.3181 x (WBC/100) + 0.6322 x (haemoglobin/100) – 0.0647 x (blasts + 1) – 0.4171 x [(spleen + 0.1) /10]	Low risk ≥0.4911 Intermediate risk: −0.8413–<0.4911 High risk: <−0.8413	Low risk: *n *= 148, 17% Intermediate risk: *n *= 506, 59% High risk: *n *= 198, 24%	PMID: 35650426
Treatment failure TKI (Zhang) [38]	0.1919 [if sex = male] + 1.6160 × (age/100) + 0.3105 × (haemoglobin concentration/100)^−2^ + 0.1087 × blasts + 0.0671 × spleen + 0.5461 [if high-risk ACA in Ph+ cells]	Low risk ≤1.3115 Intermediate risk: >1.3115–2.4266 High risk: >2.4266	Low risk: *n *= 716, 41% Intermediate risk: *n *= 812, 46% High risk (*n *= 234, 13%)	PMID: 39046786

*EUTOS* European Treatment and Outcome Study, *ELTS* EUTOS Long-Term Survival, *IMTF* Imatinib Treatment Failure, *TKI* Tyrosine Kinase Inhibitor, *WBC* White Blood Cell Count, *ACA* Additional Chromosomal Abnormalities, *Ph+* Philadelphia-positive.

^a^The distribution refers to the training set of the original publication.

The Euro score [17] followed the Sokal score in the 1990s. It was developed based on a cohort of 908 interferon-treated patients from across Europe and validated using an independent cohort of 493 patients. Like the Sokal score, the Euro score is calculated at the time of diagnosis and categorizes patients into three risk groups regarding overall survival. The Euro score is also prognostically useful for Hydroxyurea and demonstrated a predictive component, as improved survival under Interferon compared to Hydroxyurea could be expected in the low- and intermediate-risk groups [18]. Similar to the Sokal score, the Euro score incorporates age, spleen size, platelets, and blasts, with the additional inclusion of eosinophils and basophils.

With the introduction of Imatinib and the associated dramatic improvement in survival for CML patients, the need for a new prognostic tool became increasingly evident. This led to the development of the European Treatment and Outcome Study (EUTOS) Score in 2011 by Hasford et al. [19]. The focus on achieving a complete cytogenetic response (CCyR) within 18 months and progression-free survival as target outcomes reflects the evolving goals of CML therapy. This score, too, is calculated solely at the time of diagnosis. Based only on basophil counts and spleen size, the EUTOS score differentiates between just two risk groups. It was developed using data from 926 Imatinib-treated patients from the European EUTOS Registry and validated with cohorts of 616 and 1190 patients, respectively. Additional validations have been conducted by international research groups [20–23].

In 2016, the EUTOS Long-Term Survival (ELTS) Score was introduced. Pfirrmann et al. [24] developed this score using a cohort of 2205 predominantly imatinib-treated patients, some of whom had already been included in the development of the original EUTOS score. The target metric of this score is survival specifically related to CML, with CML-related death defined by prior progression of the disease. The analysis accounted for competing events, with non-CML-related death being classified as such. The ELTS score categorizes patients into three risk groups and is calculated based on age, platelet count, spleen size, and blast percentage, all of which are assessed at the time of diagnosis. This score has already been validated by other research groups [25–28].

Lauseker et al. [29] demonstrated that the score has prognostic value even for patients presenting with de novo advanced-phase CML, as it was differentiating between high- and non-high-risk patients. Additionally, it has been shown that the ELTS score holds prognostic value in children for predicting PFS [30, 31]. In contrast no prognostic value for the Euro and Sokal scores in paediatric populations was found [32].

In 2022, Zhang et al. [33] published a score designed to predict treatment failure under Imatinib therapy, based on the 2020 ELN classification. Unlike previous scores, this model focused on failure-free survival as its primary endpoint. The Imatinib Treatment Failure (IMTF) Score was developed using data from 1364 patients at Peking University People’s Hospital who were initially treated with Imatinib. The dataset was split into a 2:1 ratio for training and validation, enabling internal validation. Additional validation was conducted on a separate patient cohort from the same hospital [34]. Like other scores, the IMTF score is calculated at the time of CML diagnosis. It is based on the categorization provided by the ELTS score [24], along with haemoglobin levels, white blood cell count (WBC), and basophil count. Patients are divided into five risk groups. A first attempt to validate the score outside of China was undertaken by an Italian research group, [35]. It was however only partially successful, see the discussion.

Using the same patient cohort, Zhang et al. [36] developed two scores aimed at predicting molecular remissions in CML patients treated with Imatinib at the time of CML diagnosis. These scores are designed to predict major molecular remission (MMR) or MR^4^ (*BCR::ABL1*  ≤  0.01% IS) and focus more on future therapeutic decisions, such as the potential cessation of treatment. Nevertheless, these scores cannot be strictly classified as predictive, as no differences between treatments were considered in the risk groups. Both models use – with varying weights – gender, WBC, haemoglobin levels, blasts in peripheral blood, and spleen size below the costal margin. Unfortunately, no concise name for these scores has been established yet. Validation was performed on an additional 2184 Chinese patients [37].

Recently, a new score, again developed by Zhang et al. [38], was published. This score extends the previous IMTF score [33] to predict treatment failure for additional TKIs. This score uses gender, age, haemoglobin, spleen size, blasts in peripheral blood, and the presence of high-risk additional chromosomal abnormalities (ACAs) in Philadelphia-positive cells to classify patients into three groups. The score was developed using 1955 patients from Peking University People’s Hospital, likely with some overlap in the patient cohort. Validation was performed on a total of 3454 patients from other Chinese centres. The authors emphasize that further validation in an external patient cohort outside China would be desirable, though this has not yet been achieved, due to the recent publication.

A special category of prognostic scores applies to haematopoietic stem cell transplantation (HSCT). Unlike the previously mentioned scores, these are calculated at the time of transplantation. The Hematopoietic Cell Transplantation-Comorbidity Index (HCT-CI) [39–41] and the Disease Risk Index (DRI) [42, 43] are not specific to CML, but can be applied to any allogeneic HSCT. The HCT-CI focusses on comorbidities, similar to the Charlson Comorbidity Index [44], while the DRI categorizes haematologic malignancies based on disease type and stage. There is however one score, specifically designed for CML (but later also extended to other diseases), the well-known European Group for Blood and Marrow Transplantation (EBMT) risk score [45]. Gratwohl et al. distinguished five groups with regard to overall survival and transplant-related mortality, defined by donor type, stage of disease, age of recipient, recipient-donor combination and time from diagnosis to transplantation. The score has been validated by several groups [46–48] and is still in use, though the role of HSCT in CML has diminished with the introduction of TKI.

## Comparisons of scores

Comparisons of the various CML scoring systems have been conducted over recent years. In 2020, Pfirrmann et al. [49] compared the ELTS score with the Sokal, Euro, and EUTOS scores in a cohort of 5154 predominantly Imatinib-treated patients across Europe. Their findings indicated that the Sokal score significantly overestimates the size of the high-risk group, while the ELTS score proved prognostically superior to the other three scores in terms of both CML-specific and overall survival.

Zhang et al. [50] also evaluated the prognostic performance of the ELTS score compared to the Sokal score in a population of 1661 Chinese patients primarily treated with TKIs. In addition to survival and CML-specific survival, they analysed outcomes such as CCyR, MMR, MR^4^, MR^4.5^ (*BCR::ABL1*  ≤  0.0032% IS), FFS, and PFS - outcomes for which both scores had not been designed for. Their research showed that the ELTS score was superior to the Sokal score in predicting MR^4^, MR^4.5^, and CML-specific survival among patients treated with first-line Imatinib. For patients receiving second-generation TKIs as first-line therapy, the ELTS score outperformed the Sokal score in predicting CCyR, MMR, MR^4^, FFS, and PFS.

Brecchia et al. [51] showed in a cohort of 1206 Italian patients receiving imatinib, dasatinib or nilotinib that the ELTS score was superior to the Sokal score with regard to overall survival.

Similar results were observed in a study by Iriyama et al. [52], who analysed 610 Japanese patients from clinical trials. They assessed progression-free survival, overall survival, and CML-specific survival in cohorts treated with Imatinib or second-generation TKIs. Their findings confirmed the superiority of the ELTS score over the Sokal score in both treatment groups. In earlier research involving what was likely an overlapping cohort, the same group had demonstrated the ELTS core’s superiority over the Sokal, Euro, and EUTOS scores in predicting overall survival [25]. It has however to be stated that both publications estimated cumulative incidences for CML-specific death using Kaplan-Meier curves instead of considering competing events.

In general, validation should primarily focus on the endpoint(s) for which the authors of the score claimed that their prognostic model would work for. Since the ELTS score was developed to discriminate probabilities of “death due to CML”, statistically significant and clinically relevant differences between the score’s risk groups should be identified for this endpoint using an appropriate validation sample. In addition, though not specifically optimized for it, the creators of the ELTS score stated that it would also perform with respect to OS probabilities considering any type of death. For any other endpoint, reasonable risk group discrimination was neither claimed nor can it be guaranteed. Finally, the term “appropriate validation sample” encompasses not only an adequate definition through in- and exclusion criteria but also, in general, the sample size. For time-to-event endpoints like probabilities of dying or surviving, rather the number of events matters. Obviously, small patient samples and short follow-up times are opposed to the probability to observe a sufficient number of events. It is, therefore, inappropriate to report the failure of a prognostic model if, in the validation attempt, the number of events within the risk groups was too low [53].

These findings from the score comparisons align with current recommendations from the European LeukemiaNet (ELN) [54, 55], which advocate the use of the ELTS score. In contrast, the still relatively widespread Sokal score remains relevant solely in a scientific context, where it is needed on rare occasions to compare historical controls. However, it should no longer be employed for the prognostic classification of patients diagnosed in 2025. The same applies to the Euro score: while it is still capable of distinguishing prognostic groups, its effectiveness is suboptimal, as it generally cannot match the discriminative power of more modern scoring systems. Furthermore, the Euro score requires the largest number of parameters among all currently available scores, which is a significant drawback. The EUTOS score represents a unique case. Although the patient population on which it is based remains relatively current, its endpoints—complete cytogenetic response and progression-free survival —have substantially diminished in clinical relevance over time.

Regarding treatment-free survival with TKIs, Zhang et al. [38] demonstrated the superiority of their score compared to the ELTS and Sokal scores. The authors themselves noted that this finding is unsurprising, as the score was specifically optimized for this endpoint.

However, it remains too early to fully assess the score’s utility, as well as that of other scores introduced during the 2020 s. Should these newer scores be validated using international patient cohorts, they are likely to gain wider adoption in the future and could be applied to predict outcomes for their respective parameters.

From a methodological point of view, the use of therapy failure as an endpoint presents challenges. This is because it aggregates diverse events that can vary significantly in severity. Additionally, not all individual components are consistently recorded for every patient, and differences in definitions across countries and study groups can further complicate comparability. This problem has been described in detail by Pfirrmann et al. [14]. That said, therapy failure is recognized as the most clinically relevant criterion for assessing disease progression and for making treatment decisions [56]. Currently, a general prognosis under any TKI therapy is considered more important than one focused solely on therapy failure under Imatinib. This suggests that the most recently introduced score [38] may eventually surpass the IMTF score in utility.

## Outlook

It will be interesting to see what developments emerge in the future for prognostic research in CML. Some trends, however, already point toward potential directions.

There is a range of TKIs available that differ in their response to specific mutations and in their side effect profiles. These factors—alongside health-economic considerations and availability in a given country—are usually decisive for the choice of TKI. When the TKI is selected based on the mutation profile, there are currently no known differences in overall survival between these treatments. Under present conditions, it would be nearly impossible to demonstrate such differences statistically. This implies that a truly predictive score, one capable of highlighting differences in outcomes depending on the chosen therapy, is highly unlikely. The most practical application for such a truly predictive score would be in predicting molecular therapy responses, especially in preparation for a treatment discontinuation attempt. The score developed by Zhang et al. [36] is strictly speaking a prognostic score; if it also demonstrates predictive capabilities, this would be a welcome — albeit unintended — additional benefit.

Interestingly, all prognostic scores developed since the Sokal score rely on relatively similar markers, which have the advantage of being straightforward to measure. The only notable exception is the inclusion of high-risk ACAs in Zhang’s new score for predicting therapy failure [38]. High-risk ACAs are well-established prognostic markers [57], but they affect only a small patient group (<4% in the original study) and require cytogenetic testing. If such data are unavailable or unusable, the score cannot be calculated. However, when these data are reliably available, they can provide valuable insights. The incorporation of cytogenetic or molecular markers is already standard in other haematological disorders, such as myelodysplastic neoplasms [58, 59]. In CML, however, this practice has not yet been widely adopted. One reason could be the greater homogeneity of CML as a disease, and another might be the relatively low frequency of specific aberrations or mutations, which complicates statistical analysis. Nevertheless, it is conceivable that future scores will increasingly utilize these types of information as the field evolves.

A key focus for future scores may be their international applicability. All the current scores were developed either on entirely European or entirely Chinese populations. The difficulties of transferring these results to other populations are highlighted by the attempt to validate the IMTF score by Ielo et al. [35]. In the original population, around 8% were classified as very high risk and 16% as high risk. However, in Ielo et al.‘s Italian cohort, these categories combined accounted for less than 2%. This clearly illustrates that results are not necessarily globally transferable. The reasons for this can vary, ranging from potential ethnic differences to variations in healthcare systems. In particular, differences in the availability of therapies (both generally and for specific population groups) and surveillance practices are important factors. For the development of future scores, international collaboration would be highly desirable, despite the challenges involved.

One common feature of all CML scores is that they are calculated at a specific time, usually at the time of diagnosis. Over the course of treatment, this initial assessment naturally loses relevance. Treatment progress is then monitored via predefined milestones. These milestones provide assessments at predefined points during treatment to determine whether the patient is still “on the right track” [60–62]. In the future, dynamic predictions might be possible, which would adjust the patient’s prognosis based on each examination throughout the course of therapy [63]. The development of such a score would, despite its methodological challenges, allow for continuous patient assessment. However, the calculation would likely be much more complex and would no longer be done manually by the individual physician. This raises the question of how urgently such a score is needed, as CML is generally very well treatable today, and outliers are rare.
